# Azithromycin Resistance Patterns in *Escherichia coli* and *Shigella* before and after COVID-19, Kenya

**DOI:** 10.3201/eid3014.240374

**Published:** 2024-11

**Authors:** Elizabeth A. Odundo, Erick C. Kipkirui, Margaret C. Koech, Mary C. Kirui, Ronald K. Kirera, Nancy C. Kipkemoi, Janet N. Ndonye, Alex Ragalo, Collins K. Kigen, James W. Muturi, Vanessa N. Onyonyi, Gathii Kimita, Erick K. Muthanje, Marissa K. Hetrich, Evelyn W. Mahugu, Kirti K. Tiwari, Hunter J. Smith

**Affiliations:** Kenya Medical Research Institute, Kericho and Kisumu, Kenya (E.A. Odundo, E.C. Kipkirui, M.C. Koech, M.C. Kirui, R.K. Kirera, N.C. Kipkemoi, J.N. Ndonye, A. Ragalo, C.K. Kigen, J.W. Muturi, V.N. Onyonyi, G. Kimita, E.K. Muthanje); Walter Reed Army Institute of Research–Africa, Kericho and Kisumu (E.A. Odundo, E.C. Kipkirui, M.C. Koech, M.C. Kirui, R.K. Kirera, N.C. Kipkemoi, J.N. Ndonye, A. Ragalo, C.K. Kigen, J.W. Muturi, V.N. Onyonyi, G. Kimita, E.K. Muthanje, K.K. Tiwari); Cherokee Nation Strategic Programs, Silver Spring, Maryland, USA (M.K. Hetrich); Armed Forces Health Surveillance Division, Silver Spring (M.K. Hetrich, E.W. Mahugu, H.J. Smith); General Dynamics, Silver Spring (E.W. Mahugu)

**Keywords:** Azithromycin, *Escherichia coli*, *Shigella*, enteric infections, bacteria, antimicrobial resistance, infectious diarrhea, fluoroquinolone, antibiotics, COVID-19, Kenya

## Abstract

*Escherichia coli* and *Shigella* spp. are leading bacterial causes of acute diarrhea in sub-Saharan Africa and pose risks to global communities, travelers, and the US military. Increasing antimicrobial resistance (AMR) in those and other enteric pathogens creates treatment challenges for clinicians. Inappropriate use of antimicrobial drugs, such as azithromycin for viral respiratory infections, increased during the COVID-19 pandemic. We evaluated AMR trends of 116 *E. coli* and 109 *Shigella* spp. isolates obtained from 1,672 pre–COVID-19 (2017–2019) and 1,118 post–COVID-19 (2022–2023) human fecal samples from Kenya. Azithromycin resistance increased significantly from before to after COVID-19, from 6.3% to 40.4% (p = 0.001). Phenotypic AMR profiles from a subset of isolates were compared with genotypic AMR information derived from whole genome sequencing. The most common AMR gene detected was the macrolide *mph(A)* gene. This study highlights the need for continued AMR surveillance.

Enteric infections are preventable and treatable but remain a leading cause of illness and death globally by causing >1.6 million fatalities overall and >525,000 deaths in children <5 years old in low- and middle-income countries, such as Kenya, each year (*1*). Although diarrheal illnesses are typically self-limiting, antimicrobial treatment for bacterial enteric infections is used to reduce the duration and severity of symptoms and to prevent other severe illnesses and long-term sequelae (*2*–*4*). However, the growing global public health threat of antimicrobial resistance (AMR) is a major challenge in treating illnesses such as bacterial enteric infections (*2*–*4*).

Bacterial enteric infections are relevant to the US military because they can cause outbreaks and limit service members’ abilities to work effectively. Bacterial enteric infections are consistently the number 1 infectious disease threat according to the Military Infectious Disease Research Program’s threat prioritization panel (*4*). Therefore, effectively treating bacterial enteric infections is critical. Conducting surveillance to understand the epidemiology of diarrheal illness and AMR patterns among bacterial causes supports military and global health objectives to combat AMR and diarrheal illness (*3*).

Improper use of antimicrobial drugs can reduce bacterial susceptibility and contribute to AMR (*5*,*6*). Kenya faces major challenges in regulating antimicrobial access and use that were substantially exacerbated during the COVID-19 pandemic (*7*–*14*). The ease of access to antimicrobial drugs in many countries is a contributing factor to AMR (*15*). Furthermore, few published studies have described AMR patterns in enteric bacteria in Kenya. We investigated the potential effects of the COVID-19 pandemic on azithromycin and fluoroquinolone resistance in *Escherichia coli* and *Shigella* spp. isolates from enteric infections collected across various sites in Kenya before (2017–2019) and after (2022–2023) the COVID-19 pandemic.

## Methods

### Case Definition

*E. coli* and *Shigella* spp. isolates were recovered from participants in Kenya who had symptomatic diarrheal illness. Participants were recruited from county hospital surveillance sites including Busia, Kericho, Kisii, Kisumu, Kombewa, Uasin Gishu, and Lamu counties (Appendix Figure 1).

### Enrollment Strategy

Study staff enrolled participants during 2 time periods: March 2017–December 2019 (pre–COVID-19) and January 2022–May 2023 (post–COVID-19). Persons who sought outpatient care for acute diarrheal illness and were willing to provide a fecal specimen were enrolled regardless of sex, age, or military status. A standard questionnaire was used to collect participant information. All participants consented to their inclusion in this study.

### Process for Isolation and Selection

We plated fecal specimens on hektoen enteric, MacConkey, and MacConkey sorbitol agars (BD Diagnostic Systems, https://www.bd.com) and incubated the cultures overnight aerobically at 37°C to recover lactose fermenting (*E. coli*) and non–lactose- and non–sorbitol-fermenting (*Shigella* spp.) colonies. We conducted bacterial identification and antimicrobial susceptibility testing (AST) of suspected *E. coli* and *Shigella* spp. isolates by using the MicroScan WalkAway (Beckman Coulter, https://www.beckmancoulter.com), the Phoenix automated microbiology system (BD Diagnostic Systems), and Etest strips (bioMérieux, https://www.biomerieux.com). We cultured *E*. *coli* isolates for confirmatory PCR testing and extracted the isolate DNA by boiling at 100°C. We performed multiplex PCR (Appendix Table 1) by using the Veriti thermocycler (Thermo Fisher Scientific, https://www.thermofisher.com) and a 2% agarose gel (Millipore Sigma, https://www.sigmaaldrich.com), and gel documentation by using iBright 1000 (Thermo Fisher Scientific). 

### Antimicrobial Susceptibility Testing

We performed AST for ciprofloxacin and levofloxacin on all *E. coli* and *Shigella* spp. isolates by using the MicroScan WalkAway gram-negative NC66 panels (Beckman Coulter) for pre–COVID-19 isolates and the Phoenix gram-negative panels (BD Diagnostic Systems) for post–COVID-19 isolates. We interpreted MICs in accordance with Clinical and Laboratory Standards Institute (CLSI) guidelines (*16*). We conducted additional AST with azithromycin Etest strips (bioMérieux) by using the manufacturer’s instructions on Mueller Hinton agar plates (BD Diagnostic Systems) and incubating at 37°C for 16–20 hours. We used *E. coli* ATCC 25922 as the quality control strain for each day of testing. We selected isolates for sequencing on the basis of resistance to either azithromycin or fluoroquinolones of interest (ciprofloxacin and levofloxacin).

### Genomic DNA Extraction

We extracted DNA from the *E. coli* and *Shigella* spp. isolates by using QIAmp Fast DNA Stool Mini Kit (QIAGEN, https://www.qiagen.com) according to the manufacturer’s instructions. We quantified DNA concentrations by using Qubit 4 and the Qubit 1X dsDNA High Sensitivity assay kit (Thermo Fisher Scientific). We stored the DNA at –20°C before sequencing.

### Genomic Sequencing

We prepared DNA libraries by using Nextera XT DNA Library Preparation Kit (Illumina, https://www.illumina.com) according to the manufacturer’s instructions. We ran the library on a TapeStation 4200 (Agilent Technologies, https://www.agilent.com) to determine its average length and quality. We sequenced a 750-pM library spiked with 10% Phix on the NextSeq 2000 (Illumina) by using the P1 (300 cycles) paired end reagents (Illumina).

### Bioinformatics Analysis

We assessed the quality of the raw reads by using FastQC (*17*). We trimmed the low-quality reads, string of Ns, and adaptor sequences by using fastp (*18*). We performed genome assembly by using Shovill (https://github.com/tseemann/shovill), and we assessed genomic features (e.g., genome size, number of contigs N50) by using QUAST (*19*). We screened for antimicrobial resistance and virulence genes by using abritAMR (*20*), whereas we screened the plasmid replicons by using ABRicate (https://github.com/tseemann/abricate) against the PlasmidFinder database (*21*). We determined sequence types by using MLST version 2.23.0 (https://github.com/tseemann/mlst), phylogroup by EzClermont (*22*), and fim types by FimTyper version 1.1 (*23*). We determined the *Shigella* spp. cluster type, serotype, and O and H antigens by using ShigEiFinder (*24*). Finally, we generated a maximum-likelihood single-nucleotide polymorphism–based core genome phylogenetic tree by using Parsnp (*25*) (*Shigella* spp. reference sequence GCF_000022245.1 and *E. coli* reference sequence GCF_000005845.2) and annotated on iTOL (*26*).

### Analytic Methods

We interpreted the results of the phenotypic analysis according to CLSI standards (*16*). We pooled data across sites after confirmation of outcome homogeneity (Appendix Figure 2). Acute diarrhea was defined in this study as 3–5 loose stools over a 24-hour period and severe acute diarrhea as >5 stools over a 24-hour period. When comparing resistance over time, we grouped partially resistant intermediate isolates with fully resistant isolates. We assessed the differences in antimicrobial resistance levels pre–COVID-19 and post–COVID-19 by using a 2-tailed Fisher exact test at significance level of 0.05 and analyzed by using R Statistical Software version 4.3.1 (The R Project for Statistical Computing, https://ww.r-project.org). No adjustments were made for multiple observations.

## Results

### Sample Collection and Case Identification

During a 4.5-year period in Kenya, 2,790 fecal samples were collected and tested for *E. coli* and *Shigella* spp. Of those, 1,672 (59.9%) specimens were collected 3 years before the COVID-19 pandemic (March 2017–2019). Adult patients >18 years old provided 767 (45.9%) of the pre–COVID-19 samples and 905 (54.1%) were from children. The remaining 1,118 (40.1%) samples were collected during the 1.5-year period after the COVID-19 pandemic (January 2022–May 2023). Adult patients provided 679 (60.7%) samples and 439 (39.3%) samples came from children. We identified 116 *E. coli* isolates in total, 75 (64.7%) from children (44 [58.7%] pre– and 31 [41.3%] post–COVID-19) and 41 (46.3%) from adults (19 [46.3%] pre– and 22 [53.7%] post–COVID-19). We identified 109 *Shigella* spp. isolates in total, 57 (52.3%) from adults (31 [54.4%] pre– and 26 [45.6%] post–COVID-19) and 52 (47.7%) from children (38 [73.1%] pre– and 14 [26.9%] post–COVID-19) (Table 1).

**Table 1:** Characteristics of *Escherichia coli* and *Shigella* cases in Kenya before (2017–2019) and after (2022–2023) COVID-19*

Characteristic	*E. coli*, n = 116	*Shigella*, n = 109	p value†
Median age, y (interquartile range)	7 (3–25)	18 (4–28)	0.02
Age group			0.01
Children <18 y	75 (64.7)	52 (47.7)	
Adults >18 y	41 (35.3)	57 (52.3)	
Study period			0.17
Pre–COVID-19, 2017–2019	63 (54.3)	69 (63.3)	
Post–COVID-19, 2022–2023	53 (45.7)	40 (36.7)	
County site			0.96
Busia County Referral Hospital	18 (15.5)	15 (13.8)	
Kericho County Referral Hospital	40 (34.5)	38 (34.9)	
Kombewa County Hospital	10 (8.6)	7 (6.4)	
Kisii Teaching and Referral Hospital	30 (25.9)	31 (28.4)	
Uasin Gishu	17 (14.7)	18 (16.5)	
Lamu	1 (0.9)	0	
Diarrhea severity			0.75
No acute diarrhea	3 (2.6)	1 (0.9)	
Acute diarrhea‡	53 (46.5)	50 (45.9)	
Severe acute diarrhea§	58 (50.9)	58 (53.2)	
Water source§			
Municipal	54 (47.0)	56 (51.4)	0.51
Rain	22 (19.1)	30 (27.5)	0.14
Borehole	22 (19.1)	18 (16.5)	0.61
Spring	9 (7.8)	18 (16.5)	0.046
Well	7 (6.1)	11 (10.1)	0.27
Bottle	3 (2.6)	4 (3.7)	0.72
Tap	1 (0.9)	0	>0.99
Stream	1 (0.9)	0	>0.99
Other	0	1 (0.9)	0.49
Water treatment¶			
No treatment	81 (70.4)	74 (67.9)	0.68
Boil	20 (17.4)	22 (20.2)	0.59
Distillation	0	1 (0.9)	0.49
Chemical	1 (0.9)	1 (0.9)	>0.99
Chlorine	0	1 (0.9)	0.49
Water guard	14 (12.2)	10 (9.2)	0.47
Ciprofloxacin susceptibility (*15*)			0.25
Susceptible	110 (97.3)	108 (100)	
Intermediate	0	0	
Resistant	3 (2.7)	0	
Levofloxacin susceptibility (*15*)			0.25
Susceptible	110 (97.3)	108 (100)	
Intermediate	0	0	
Resistant	3 (2.7)	0	
Azithromycin susceptibility (*15*)			<0.001
Susceptible	89 (76.7)	105 (96.3)	
Intermediate	1 (0.9)	0 (0)	
Resistant	26 (22.4)	4 (3.7)	

*Values are no. (%) except as indicated. One adult *E. coli* case was missing data and removed from denominators for the following variables: diarrhea severity, water source, and water treatment. Four cases (3 *E. coli* and 1 *Shigella*) had inconclusive ciprofloxacin and levofloxacin susceptibility results and were excluded from susceptibility profiles. Any missing data were excluded from analysis. †We obtained p values by using the Wilcoxon rank sum test, Fisher exact test, or Pearson χ^2^ test, as appropriate. ‡Defined as 3–5 loose stools over a 24-h period. §Defined as >6 loose stools over a 24-h period. ¶Participants could select multiple water sources or treatments.

### Demographics and Prevalence of Cases

Across both study periods, *E. coli* case-patients were on average younger than *Shigella* spp. case-patients (median [interquartile range] 7 years [3–25] vs. 18 [4–28] years of age; p = 0.02). Nearly all *E. coli* (97.4%) and *Shigella* spp. (99.1%) case-patients reported either acute or severe acute diarrhea. Most *E. coli* (61.8%) and *Shigella* spp. Case-patients were from Kericho and Kisii. Municipal water was the most frequently reported water source among both *E. coli* and *Shigella* spp. case-patients, followed by rain, boreholes, and spring water. Water treatment was uncommon; only 30.8% of all case-patients reported chemical or physical water treatment methods, which did not meaningfully vary by pathogen (Table 1). Overall, *E. coli* cases increased from 3.8 (95% CI 2.9–4.8) per 100 persons pre–COVID-19 to 4.7 (95% CI 3.5–6.2) per 100 persons post–COVID-19 (p = 0.21). Of note, recovery of *E. coli* isolates from children increased from 4.9 (95% CI 3.6–6.5) per 100 persons pre–COVID-19 to 7.1 (95% CI 4.9–9.9) per 100 persons in the post–COVID-19 period (p = 0.10). Among adults, there was a slight increase from 2.5 (95% CI 1.5–3.8) to 3.2 (95% CI 2.0–4.9) per 100 persons (p = 0.38). *Shigella* spp. prevalence remained steady across COVID-19 periods and age groups, ranging from 3.2 to 4.2 per 100 persons (Table 2).

**Table 2:** Cases and antimicrobial susceptibility of Escherichia coli and Shigella before (2017–2019) and after (2022–2023) COVID-19, Kenya*

Characteristic	*E. coli*					*Shigella* spp.			
	Overall	2017–2019	2022–2023	p value†		Overall	2017–2019	2022–2023	p value†
All ages									
No. cases	116	63	53	NA		109	69	40	NA
No. tested	2,790	1,672	1,118	NA		2,790	1,672	1,118	NA
Cases/100 persons (95% CI)	4.2 (3.5–5.0)	3.8 (2.9–4.8)	4.7 (3.5–6.2)	0.21		3.9 (3.2–4.7)	4.1 (3.2–5.2)	3.6 (2.6–4.8)	0.47
% Resistant isolates‡									
Ciprofloxacin	2.7	1.6	3.9	0.59		0	0	0	>0.99
Levofloxacin	2.7	1.6	3.9	0.59		0	0	0	>0.99
Azithromycin	23.3	7.9	41.5	**<0.001**		3.7	0	10.0	**0.02**
Adults ≥18 y									
No. cases	41	19	22	NA		57	31	26	NA
No. tested	1,446	767	679	NA		1,446	767	679	NA
Cases/100 persons (95% CI)	2.8 (2.0–3.8)	2.5 (1.5–3.8)	3.2 (2.0–4.9)	0.38		3.9 (3.0–5.1)	4.0 (2.8–5.7)	3.8 (2.5–5.6)	0.84
% Resistant isolates‡									
Ciprofloxacin	5.1	5.3	5.0	>0.99		0	0	0	>0.99
Levofloxacin	5.1	5.3	5.0	>0.99		0	0	0	>0.99
Azithromycin	24.4	5.3	40.9	**0.01**		7.0	0	15.4	**0.04**
Children <18 y									
No. cases	75	44	31	NA		52	38	14	NA
No. tested	1,344	905	439	NA		1,344	905	439	NA
Cases/100 persons (95% CI)	5.6 (4.4–6.9)	4.9 (3.6–6.5)	7.1 (4.9–9.9)	0.10		3.9 (2.9–5.0)	4.2 (3.0–5.7)	3.2 (1.8–5.3)	0.37
% Resistant isolates‡									
Ciprofloxacin	1.4	0	3.2	0.42		0	0	0	>0.99
Levofloxacin	1.4	0	3.2	0.42		0	0	0	>0.99
Azithromycin	22.7	9.1	41.9	**0.002**		0	0	0	>0.99

*Four cases (3 *E. coli* and 1 *Shigella*) had inconclusive ciprofloxacin and levofloxacin susceptibility results and were excluded from susceptibility profiles. Boldface indicates significant values (p<0.05). NA, not applicable †Pearson χ^2^ test used to measure differences in prevalence across COVID-19 periods. Fisher exact test used to measure differences in resistance proportions between the pre–COVID-19 and post–COVID-19 periods. ‡One intermediate resistant isolate grouped with fully resistant.

### Antimicrobial Resistance Patterns before and after COVID-19 

For the pre–COVID-19 period, <10% of *E. coli* isolates were resistant to ciprofloxacin (n = 1), levofloxacin (n = 1), or azithromycin (n = 4). For the post–COVID-19 period, *E. coli* resistance to ciprofloxacin, levofloxacin, or azithromycin increased to 45.1% (n = 23). This increase was predominantly because of an increase in azithromycin resistance, from 7.9% pre–COVID-19 to 41.5% post–COVID-19 (p<0.001) (Table 2). Ciprofloxacin and levofloxacin resistance increased from 1.6% to 3.9% (p = 0.59), at near identical magnitudes among both adults and children (Table 2), even when stratified by children <5 years and 5–17 years of age (Appendix Table 2). Of *Shigella* spp. isolates tested, 96.2% (n = 104) were susceptible to all 3 antimicrobial drugs. Only 4 isolates (3.7%) were resistant to only azithromycin. All 4 resistant isolates were from adults in the post–COVID-19 period (Table 2). All *Shigella* spp. isolates were susceptible to ciprofloxacin and levofloxacin (Table 2).

Similar resistance patterns were observed after excluding Busia and Lamu sites that only recruited participants in the post–COVID-19 period (Appendix Figure 2). Azithromycin resistance patterns did not significantly vary by reported water source or water treatment methods (Appendix Table 3).

### Genomic Characteristics of *E. coli* and *Shigella* spp. Isolates

**Figure 1:**
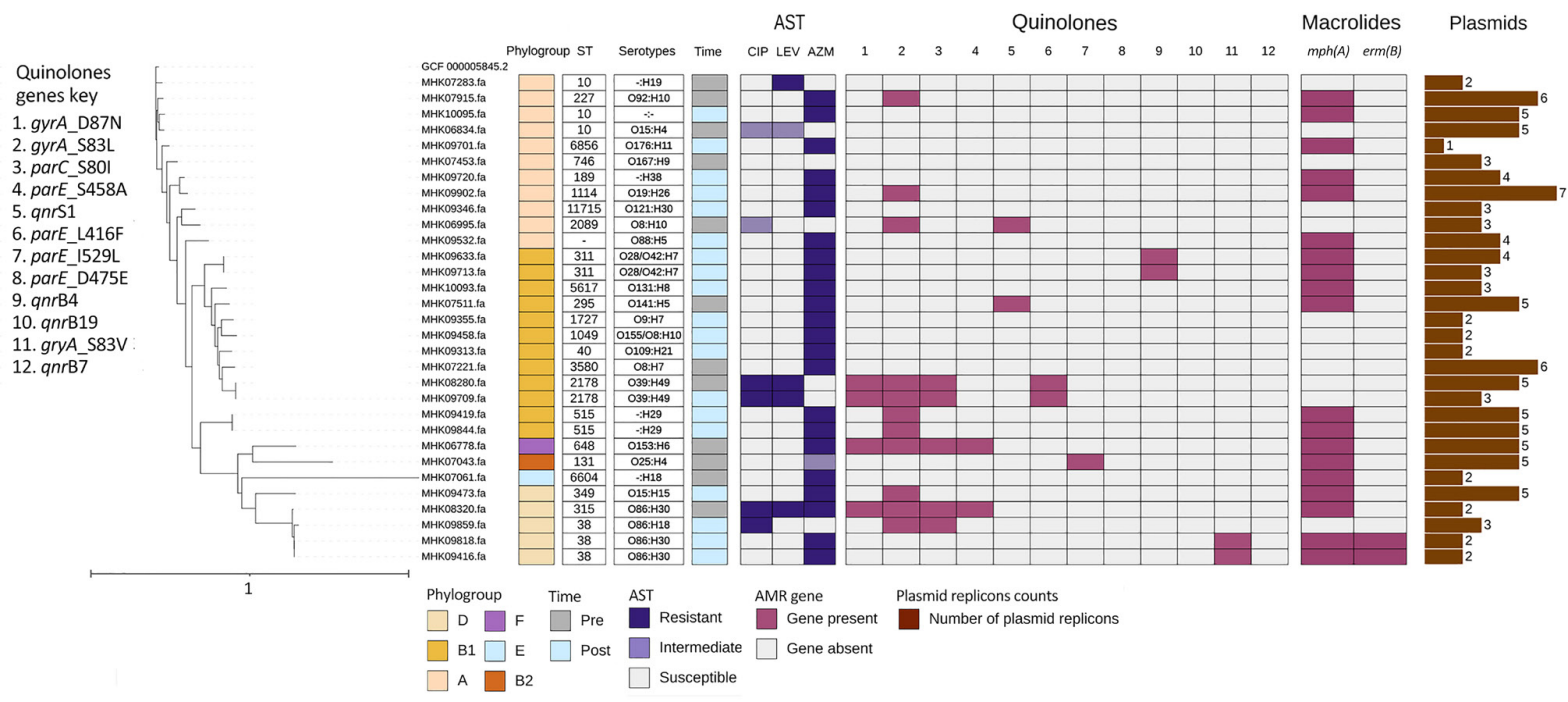
Phylogenetic tree and corresponding heatmap of 31 *Escherichia coli* isolates carrying antimicrobial resistance genes recovered from patients in Kenya with acute or severe diarrheal disease from pre–COVID-19 (2017–2019) and post–COVID-19 (2022–2023) periods. The phylogenetic tree was constructed by using a maximum-likelihood single-nucleotide polymorphism core genome alignment with a reference strain. Isolates are identified by reference genome identification numbers. Tree scale bar measures substitutions per site. AMR, antimicrobial resistance; AST, antimicrobial susceptibility testing; AZM, azithromycin; CIP, ciprofloxacin; LEV, levofloxacin; Post, post–COVID-19; Pre, pre–COVID-19; ST, sequence type.

For *E. coli* isolates, phylogenetic groups, strain types, and plasmid replicons of the 31 *E. coli* isolates characterized by whole genome sequencing (WGS) are provided in detail (Figure 1). One post–COVID-19 isolate had a missing allele and could not be identified. Macrolide resistance gene *mph(A)* (n = 19) was detected in 6/12 pre–COVID-19 and 13/19 post–COVID-19 isolates, whereas *erm(B)* was detected in 2/19 post–COVID-19 isolates. Quinolone resistance genes (n = 31) detected were *gyrA_D87N* (n = 4), *gyrA_S83L* (n = 11), *gyrA_S83V* (n = 2), *parC_S80I* (n = 5), *parE_S458A* (n = 2), *parE_L416F* (n = 2), and *parE_I529L* (n = 1). Plasmid–mediated quinolone resistance genes *qnrS1* (n = 2) and *qnrB4* (n = 2) were detected; however, only 1 isolate was not susceptible to ciprofloxacin or levofloxacin. There was also co-occurrence of both macrolide and quinolone resistance genes: *mph(A)* with *gyrA* (n = 10), *mph(A)* and *erm(B)* with *gyrA* (n = 2), and *mph(A)* with *qnrB4* (n = 2) that were also resistant to azithromycin (Figure 1).

**Figure 2:**
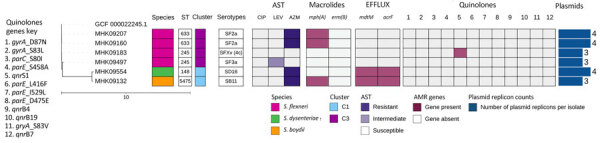
Phylogenetic tree and corresponding heatmap of 6 *Shigella* spp. isolates carrying antimicrobial resistance genes recovered from patients in Kenya with acute or severe diarrheal disease. The phylogenetic tree was constructed by using a maximum-likelihood single-nucleotide polymorphism core genome alignment with a reference strain. Isolates are identified by reference genome identification numbers. Tree scale bar measures substitutions per site. AMR, antimicrobial resistance; AST, antimicrobial susceptibility testing; AZM, azithromycin; CIP, ciprofloxacin; LEV, levofloxacin; ST, sequence type.

For *Shigella* spp., 6 isolates were characterized by WGS, belonging to 3 species: *S. flexneri* (n = 4), *S. boydii* (n = 1), and *S. dysenteriae* (n = 1). Sequence types, clusters, and serotypes are provided in detail (Figure 2). Macrolide resistance gene *mph(A)* was detected in 3/6 *Shigella* spp. isolates. All isolates phenotypically resistant to azithromycin carried the *mph(A)* gene, except 1 that carried 2 multidrug efflux pump genes, *mdtM* and *acrF*. One isolate carried a quinolone resistance gene, *qnrS1*, but its phenotypic susceptibility to ciprofloxacin and levofloxacin was inconclusive, and it was therefore excluded from AST analysis.

## Discussion

As with previous studies conducted in Kenya, acute bacterial enteric infections in this study were primarily caused by *E. coli* and *Shigella* spp. (*27*). Although *E. coli* prevalence was slightly higher in the post–COVID-19 period, the increase was not significant (p>0.05), suggesting a return to baseline circulation of enteric pathogens after the cessation of COVID-19 prevention measures. The largest increase in prevalence was observed among children <18 years of age, possibly because of the reentry of immune-naive children into public spaces and schools, as has been hypothesized for other infectious diseases.

Of note, this study revealed that *E. coli* isolates from adults and children after the COVID-19 pandemic were 5 times more likely to be resistant to azithromycin than those isolated before the pandemic. Potential explanations for those increases include Kenya’s insufficient antimicrobial regulation and suboptimal clinical use of antimicrobial drugs, particularly during the COVID-19 pandemic (*7*–*10*,*13*,*28*). The likelihood of encountering antimicrobial drugs is increased, which might enable resistance development. It is possible the increases in azithromycin resistance observed in this study were part of the gradual increase in resistance patterns over time, but it is also possible resistance patters were accelerated because of increased use of antimicrobial drugs such as azithromycin for viral respiratory infections during the COVID-19 pandemic (*12*).

Macrolide resistance genes, such as the *mph(A)* gene responsible for azithromycin resistance, are commonly found in *E. coli* (*29*). Of the isolates we sequenced, 19/31 contained macrolide resistance genes. Only 6 of those 19 *E. coli* isolates were from pre–COVID-19 samples, revealing an increase in azithromycin resistance genes after the pandemic. The *mph(A)* gene was found in different strain types, indicating the potential for transmission across *E. coli* species. Among 6 *Shigella* spp. isolates collected in the post–COVID-19 period, the 3 identified as azithromycin resistant carried the *mph(A)* gene. Literature suggests that spread of macrolide resistance genes among *Shigella* spp. is because of horizontal gene transfer rather than direct lineage (A. Asad, unpub. data, https://pubmed.ncbi.nlm.nih.gov/37461575).

Our findings are crucial for US military members who may be deployed to Kenya. *E. coli* and *Shigella* spp. infections can result in severe diarrhea and sequelae, which can reduce service members’ ability to perform expected duties. Ciprofloxacin and azithromycin can shorten symptom duration and severity and accelerate recovery from bacterial enteric infections (*6*). However, reduced antimicrobial susceptibility might impede clinician efforts to treat infections effectively and return service members back to full operational capabilities. On a global public health level, bacterial enteric pathogens can cause large outbreaks, making antimicrobial drugs critical in mitigating their negative impacts.

The first limitation of this study is that participants were enrolled as a single encounter without follow-up, so it is possible some asymptomatic participants with a pathogen later became cases. Second, there was insufficient information about residence (rural or urban) or military status, which may affect the results because of exposure differences to pathogens and antimicrobials. Third, no information was collected regarding treatment regimens and outcomes, which raised questions about the clinical effect of AMR phenotypes on patients. Fourth, the study did not include analyzable demographic data such as sex, which can influence healthcare-seeking behaviors and sensitivity to certain antimicrobial drugs. Fifth, different platforms were used for phenotypic fluoroquinolone resistance characterization before and after COVID-19 for MIC testing, which could have led to differences in AST results. However, results from both platforms were interpreted according to CLSI guidelines, limiting potential differences. Finally, data early in the COVID-19 pandemic (January 2020–December 2021) could have provided additional context to the increase in AMR, but this study was unable to capture samples during that time.

In conclusion, understanding of the AMR patterns of bacterial enteric infections, such as those observed in this study, is crucial for military and local clinicians when considering antimicrobial drugs for treating acute diarrhea. The US military must be adequately prepared to deploy into any area at any given time by understanding all potential threats, including pathogens. AMR can manifest anywhere because of globalized travel and gene transfer; therefore, continuous monitoring of phenotypic AMR and resistance gene markers against antimicrobial drugs for bacterial enteric pathogens is necessary, particularly in regions such as sub-Saharan Africa where AMR surveillance is underreported.

Appendix Additional information about azithromycin resistance patterns in *Escherichia coli* and *Shigella* before and after COVID-19, Kenya
