# Supplementary material for: Azithromycin Resistance Patterns in Escherichia coli and Shigella before and after COVID-19, Kenya
Source: Emerg Infect Dis. 2024 Nov;30(Suppl 2):S86–93. doi: 10.3201/eid3014.240374 (PMC11559570; doi:10.3201/eid3014.240374)
Supplement: Appendix — Additional information about azithromycin resistance patterns in Escherichia coli and Shigella before and after COVID-19, Kenya [file 24-0374-Techapp-s1.pdf]

Article DOI: https://doi.org/10.3201/eid3014.240374

EID cannot ensure accessibility for supplementary materials supplied by authors. Readers who have difficulty accessing supplementary content should contact the authors for assistance.

Azithromycin Resistance Patterns in *Escherichia coli* and *Shigella* before and after COVID-19, Kenya

Appendix.

Appendix Table 1. Primer sequences used in multiplex PCR for detection of diarrheagenic *Escherichia coli.*

E. coli type	Target gene	Primer sequences (5′ to 3′)	Product size in bp
ETEC	*elt*	CACACGGAGCTCCTCAGTC	508
		CCCCCAGCCTAGCTTAGTTT	
	est	GCTAAACCAGTAGAGGTCT	147
		CCCGGTACAGAGCAGGATTACAACA	
EHEC	*Stx1*	CAGTTAATGTGGTGGCGAAGG	384
		CACCAGACAATGTAACCGCTG	
	Stx2	ATCCTATTCCCGGGAGTTACG	584
		GCGTCATCGTATACACAGGAGC	
EPEC	*eae*	CCCGAATTCGGCACAAGCATAAGC	881
		CCCGGATCCGTCTCGCCAGTATTCG	
	bfpA	GGAAGTCAAATTCATGGGGGTAT	300
		GGAATCAGACGCAGACTGGTAGT	
EIEC	*ipaH*	TGGAAAAACTCAGTGCCTCT	423
		CCAGTCCGTAAATTCATTCT	
EAEC	*aatA*	CTGGCGAAAGACTGTATCAT	650
		CAATGTATAGAAATCCGCTGTT	
	aaiC	ATTGTCCTCAGGCATTTCAC	215
		ACGACACCCCTGATAAACAA	

Appendix Table 2. Antibiotic susceptibility of *E. coli* cases among children in Kenya before and after the COVID-19 pandemic.

Characteristics	Overall	Pre-COVID	Post-COVID	p-value*
Children <5 years				
No. cases	47	27	20	--
% Resistant†				
CIP	2.2%	0.0%	5.0%	0.43
LEV	2.2%	0.0%	5.0%	0.43
AZM	21.3%	7.4%	40.0%	0.01
Children 5-17 years				
No. cases	28	17	11	--
% Resistant†				
CIP	0.0%	0.0%	0.0%	>0.99
LEV	0.0%	0.0%	0.0%	>0.99
AZM	25.0%	11.8%	45.5%	0.08
All children <18 years				
No. cases	75	44	31	--
% Resistant†				
CIP	1.4%	0.0%	3.2%	0.42
LEV	1.4%	0.0%	3.2%	0.42
AZM	22.7%	9.1%	41.9%	0.002

One CIP and LEV result removed from calculations of antibiotic susceptibility in children <5 years due to an inconclusive result.
CIP = Ciprofloxacin; LEV = Levofloxacin; AZM = Azithromycin
*Pearson’s chi-square test used to measure differences in prevalence across COVID-19 periods. Fisher’s exact test used to measure difference in resistance proportions between the pre-COVID-19 and post-COVID-19 periods.
†Percent of all *E. coli* cases (No. cases column).

Appendix Table 3. Azithromycin susceptibility of *E. coli* and *Shigella* spp. isolates by water source and treatment

	E. coli			***Shigella* spp.**		
**Water source/treatment**	**Susceptible**	**Resistant**	**p-value**	**Susceptible**	**Resistant**	**p-value***
**All ages**	**N=90**	**N=26**		**N=105**	**N=4**	
**Source**						
Borehole	16 (17.8%)	6 (24.0%)	0.57	16 (15.2%)	2 (50.0%)	0.13
Rain	19 (21.1%)	3 (12.0%)	0.4	29 (27.6%)	1 (25.0%)	>0.99
Well	5 (5.6%)	2 (8.0%)	0.64	10 (9.5%)	1 (25.0%)	0.35
Bottle	3 (3.3%)	0 (0.0%)	>0.99	4 (3.8%)	0 (0.0%)	>0.99
Municipal	41 (45.6%)	13 (52.0%)	0.57	55 (52.4%)	1 (25.0%)	0.35
Spring	9 (10.0%)	0 (0.0%)	0.2	16 (15.2%)	2 (50.0%)	0.13
Stream	1 (1.1%)	0 (0.0%)	>0.99	0 (0.0%)	0 (0.0%)	>0.99
Tap	1 (1.1%)	0 (0.0%)	>0.99	0 (0.0%)	0 (0.0%)	>0.99
Other	0 (0.0%)	0 (0.0%)	>0.99	1 (1.0%)	0 (0.0%)	>0.99
**Treatment**						
Boil	15 (16.7%)	5 (20.0%)	0.77	20 (19.0%)	2 (50.0%)	0.18
Distillation	0 (0.0%)	0 (0.0%)	>0.99	1 (1.0%)	0 (0.0%)	>0.99
Chemical	1 (1.1%)	0 (0.0%)	>0.99	1 (1.0%)	0 (0.0%)	>0.99
Chlorine	0 (0.0%)	0 (0.0%)	>0.99	1 (1.0%)	0 (0.0%)	>0.99
Water guard	10 (11.1%)	4 (16.0%)	0.5	10 (9.5%)	0 (0.0%)	>0.99
No Tx	65 (72.2%)	16 (64.0%)	0.43	72 (68.6%)	2 (50.0%)	0.59
**Adults ≥18 years**						
**Source**	**N=31**	**N=10**		**N=53**	**N=4**	
Borehole	10 (32.3%)	1 (11.1%)	0.4	12 (22.6%)	2 (50.0%)	0.25
Rain	6 (19.4%)	3 (33.3%)	0.39	17 (32.1%)	1 (25.0%)	>0.99
Well	3 (9.7%)	2 (22.2%)	0.31	7 (13.2%)	1 (25.0%)	0.46
Bottle	1 (3.2%)	0 (0.0%)	>0.99	1 (1.9%)	0 (0.0%)	>0.99
Municipal	7 (22.6%)	4 (44.4%)	0.23	23 (43.4%)	1 (25.0%)	0.63
Spring	2 (6.5%)	0 (0.0%)	>0.99	8 (15.1%)	2 (50.0%)	0.14
Stream	0 (0.0%)	0 (0.0%)	>0.99	0 (0.0%)	0 (0.0%)	>0.99
Tap	1 (3.2%)	0 (0.0%)	>0.99	0 (0.0%)	0 (0.0%)	>0.99
Other	0 (0.0%)	0 (0.0%)	>0.99	0 (0.0%)	0 (0.0%)	>0.99
**Treatment**						
Boil	5 (16.1%)	1 (11.1%)	>0.99	8 (15.1%)	2 (50.0%)	0.14
Distillation	0 (0.0%)	0 (0.0%)	>0.99	1 (1.9%)	0 (0.0%)	>0.99
Chemical	0 (0.0%)	0 (0.0%)	>0.99	1 (1.9%)	0 (0.0%)	>0.99
Chlorine	0 (0.0%)	0 (0.0%)	>0.99	1 (1.9%)	0 (0.0%)	>0.99
Water guard	4 (12.9%)	0 (0.0%)	0.56	3 (5.7%)	0 (0.0%)	>0.99
No Tx	22 (71.0%)	8 (88.9%)	0.4	39 (73.6%)	2 (50.0%)	0.31
**Children <18 years**						
**Source**						
Borehole	6 (10.2%)	5 (31.3%)	**0.05**	4 (7.7%)	0 (NA%)	NA
Rain	13 (22.0%)	0 (0.0%)	0.058	12 (23.1%)	0 (NA%)	NA
Well	2 (3.4%)	0 (0.0%)	>0.99	3 (5.8%)	0 (NA%)	NA
Bottle	2 (3.4%)	0 (0.0%)	>0.99	3 (5.8%)	0 (NA%)	NA
Municipal	34 (57.6%)	9 (56.3%)	0.92	32 (61.5%)	0 (NA%)	NA
Spring	7 (11.9%)	0 (0.0%)	0.33	8 (15.4%)	0 (NA%)	NA
Stream	1 (1.7%)	0 (0.0%)	>0.99	0 (0.0%)	0 (NA%)	NA
Tap	0 (0.0%)	0 (0.0%)	>0.99	0 (0.0%)	0 (NA%)	NA
Other	0 (0.0%)	0 (0.0%)	>0.99	1 (1.9%)	0 (NA%)	NA
**Treatment**						
Boil	10 (16.9%)	4 (25.0%)	0.48	12 (23.1%)	0 (NA%)	NA
Distillation	0 (0.0%)	0 (0.0%)	>0.99	0 (0.0%)	0 (NA%)	NA
Chemical	1 (1.7%)	0 (0.0%)	>0.99	0 (0.0%)	0 (NA%)	NA
Chlorine	0 (0.0%)	0 (0.0%)	>0.99	0 (0.0%)	0 (NA%)	NA
Water guard	6 (10.2%)	4 (25.0%)	0.21	7 (13.5%)	0 (NA%)	NA
No Tx	43 (72.9%)	8 (50.0%)	0.082	33 (63.5%)	0 (NA%)	NA

Azithromycin susceptibility profile of *E. coli* and *Shigella* spp. isolates by water source and treatment, stratified by age group.
*Fisher's exact test

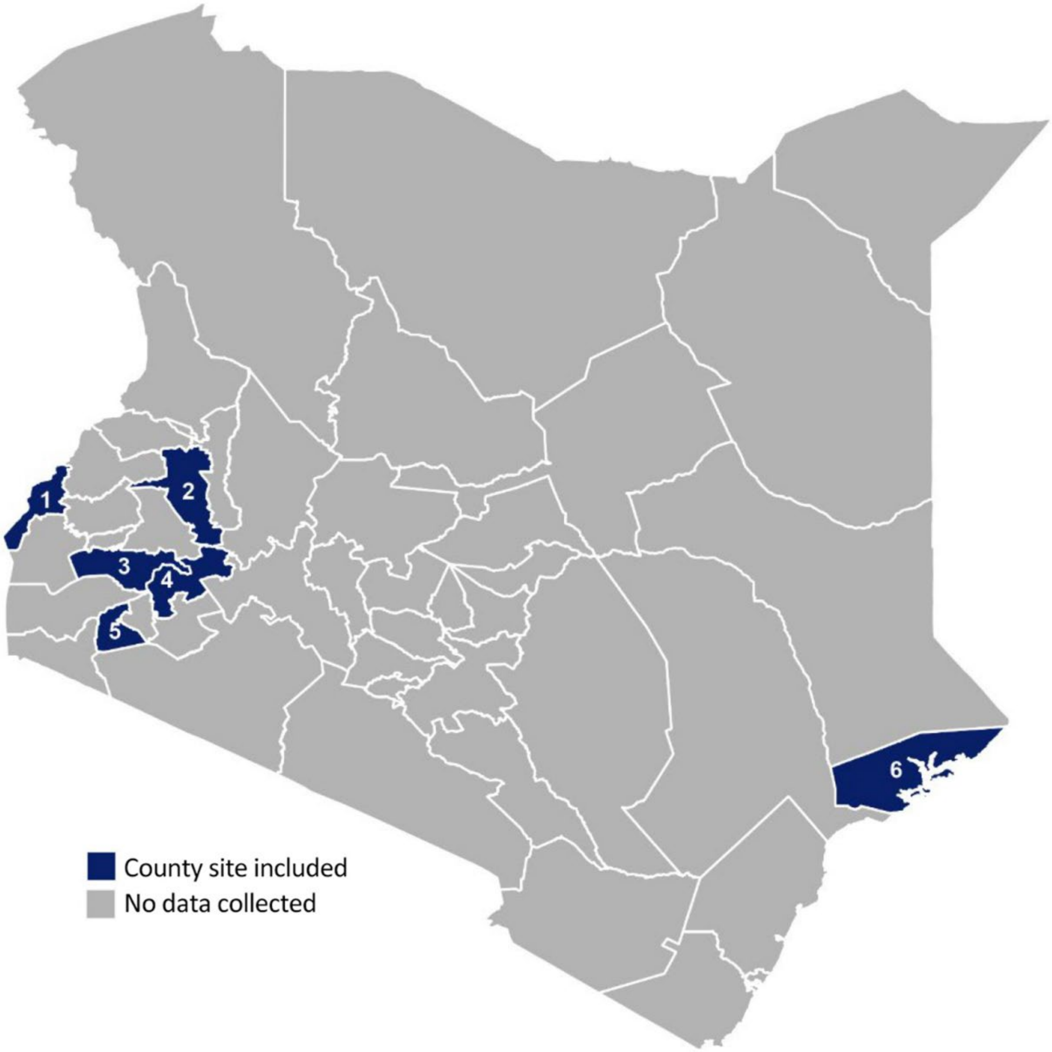

Appendix Figure 1. Map of Kenya showing the regional distribution of surveillance sites by residing county: 1) Busia, 2) Uasin Gishu, 3) Kisumu, 4) Kericho, 5) Kisii, 6) Lamu.

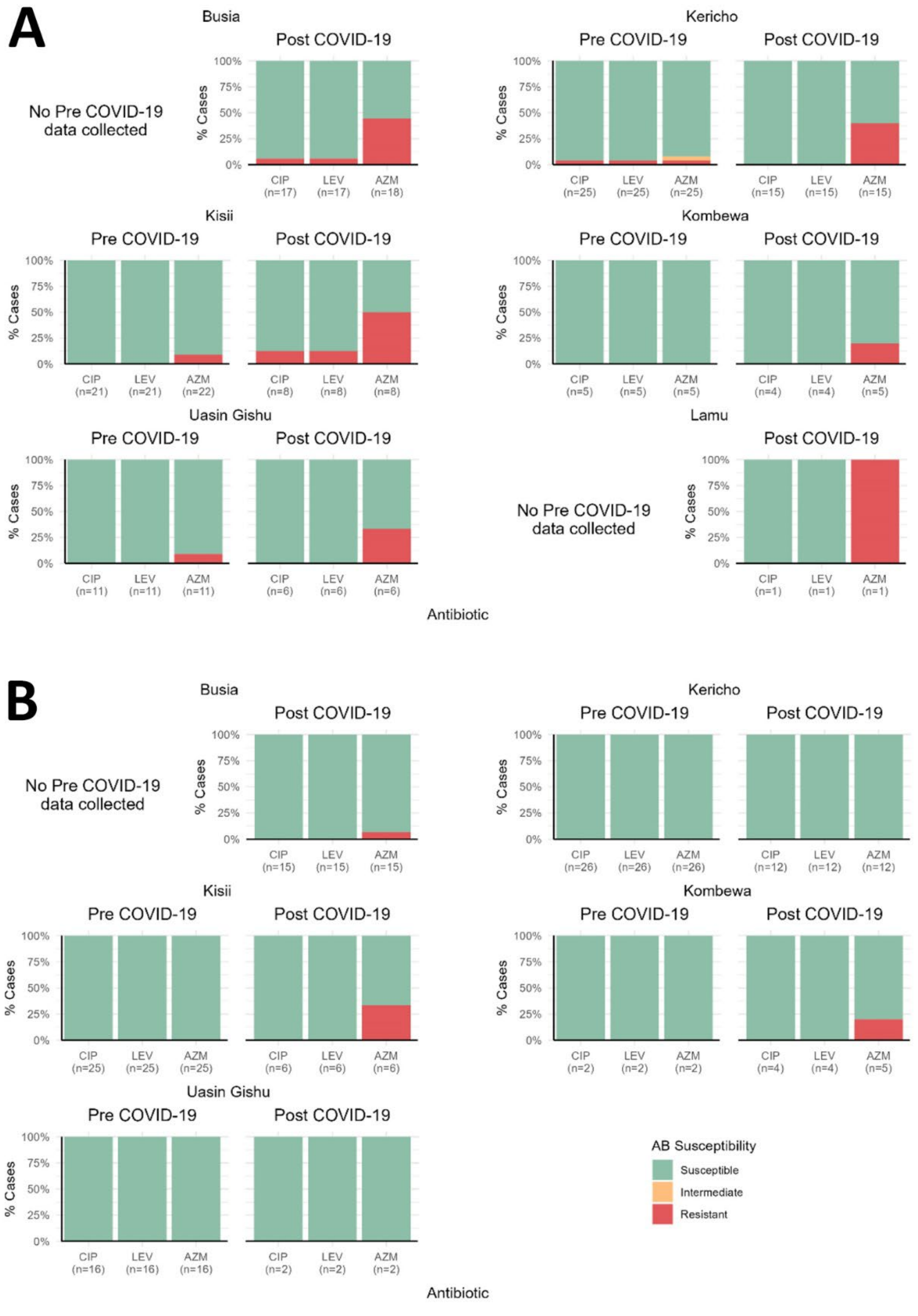

Appendix Figure 2. Antibiotic susceptibility profile of *E. coli* and *Shigella* spp. isolates by site. A) *E. coli* isolates, B) *Shigella* spp. isolates. CIP = Ciprofloxacin; LEV = Levofloxacin; AZM = Azithromycin.

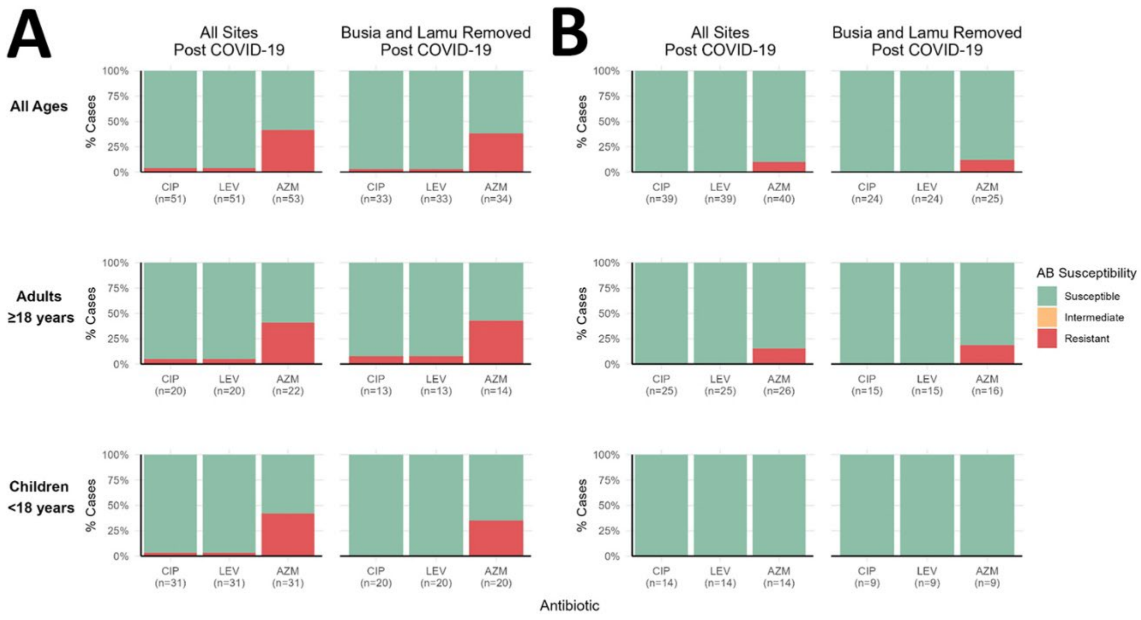

Appendix Figure 3. Post-COVID-19 antibiotic susceptibility profile of *E. coli* and *Shigella* spp. isolates excluding post-COVID-only collection sites. A) *E. coli* isolates, B) *Shigella* spp. isolates. CIP = Ciprofloxacin; LEV = Levofloxacin; AZM = Azithromycin.
